# Evidence for interhemispheric imbalance in stroke patients as revealed by combining transcranial magnetic stimulation and electroencephalography

**DOI:** 10.1002/hbm.25297

**Published:** 2021-01-13

**Authors:** Elias Paolo Casula, Maria Concetta Pellicciari, Sonia Bonnì, Barbara Spanò, Viviana Ponzo, Ilenia Salsano, Giovanni Giulietti, Alex Martino Cinnera, Michele Maiella, Ilaria Borghi, Lorenzo Rocchi, Marco Bozzali, Fabrizio Sallustio, Carlo Caltagirone, Giacomo Koch

**Affiliations:** ^1^ Non‐Invasive Brain Stimulation Unit/Department of Behavioral and Clinical Neurology Santa Lucia Foundation Rome Italy; ^2^ Department of Clinical and Movement Neurosciences UCL Queen Square Institute of Neurology, University College London London UK; ^3^ Neuroimaging Laboratory Santa Lucia Foundation Rome Italy; ^4^ Brighton and Sussex Medical School, University of Sussex Brighton UK; ^5^ Stroke Unit, Department of Neuroscience Tor Vergata Polyclinic Rome Italy

**Keywords:** electroencephalography, interhemispheric dynamics, stroke, transcranial magnetic stimulation

## Abstract

Interhemispheric interactions in stroke patients are frequently characterized by abnormalities, in terms of balance and inhibition. Previous results showed an impressive variability, mostly given to the instability of motor‐evoked potentials when evoked from the affected hemisphere. We aim to find reliable interhemispheric measures in stroke patients with a not‐evocable motor‐evoked potential from the affected hemisphere, by combining transcranial magnetic stimulation (TMS) and electroencephalography. Ninteen stroke patients (seven females; 61.26 ± 9.8 years) were studied for 6 months after a first‐ever stroke in the middle cerebral artery territory. Patients underwent four evaluations: clinical, cortical, corticospinal, and structural. To test the reliability of our measures, the evaluations were repeated after 3 weeks. To test the sensitivity, 14 age‐matched healthy controls were compared to stroke patients. In stroke patients, stimulation of the affected hemisphere did not result in any inhibition onto the unaffected. The stimulation of the unaffected hemisphere revealed a preservation of the inhibition mechanism onto the affected. This resulted in a remarkable interhemispheric imbalance, whereas this mechanism was steadily symmetric in healthy controls. This result was stable when cortical evaluation was repeated after 3 weeks. Importantly, patients with a better recovery of the affected hand strength were the ones with a more stable interhemispheric balance. Finally, we found an association between microstructural integrity of callosal fibers, suppression of interhemispheric TMS‐evoked activity and interhemispheric connectivity. We provide direct and sensitive cortical measures of interhemispheric imbalance in stroke patients. These measures offer a reliable means of distinguishing healthy and pathological interhemispheric dynamics.

## Graphical abstract

We used transcranial magnetic stimulation–electroencephalography (TMS–EEG) to characterize interhemispheric dynamics in stroke patients. We provide a direct evidence of interhemispheric imbalance in stroke patients. TMS–EEG offers a reliable assessment of cerebral state during evolution of stroke.

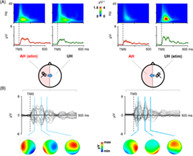

**Abbreviations**
AH: affected hemisphere
CC: corpus callosum
CST: corticospinal tract
EMG: electromyography
FA: fractional anisotropy
GPFT: grip pinch force test
HC: healthy controls
ICA: independent component analysis
ICC: intraclass correlation coefficient.
IHB: interhemispheric balance
IHCoh: interhemispheric coherence
IHI: interhemispheric inhibition
ISP: interhemispheric signal propagation
LH: left hemisphere
M1: primary motor cortex
MD: mean diffusivity
MEP: motor‐evoked potential
MNI: Montreal Neurological Institute
MRI: magnetic resonance imaging
NIHSS: national institute health stroke scale
RH: right hemisphere
RMT: resting motor threshold
TMS: transcranial magnetic stimulation
UH: unaffected hemisphere

## 1 INTRODUCTION

Poststroke alterations in cerebral excitability affect the balance between the two hemispheres, the interactions of which are crucial in the execution of unilateral and bimanual movements (Mayston, Harrison, & Stephens, 1999). Among these, the so‐called interhemispheric inhibition (IHI), i.e., the mechanism through which each hemisphere inhibits the other during unilateral movements, is of critical relevance. Indeed, during the production of voluntary unimanual movements, the fast inhibition of the motor output in the hemisphere contralateral to the moving hand is necessary to suppress mirror movements in the passive hand (Beaulé, Tremblay, & Théoret, 2012; Mayston et al., 1999), although also a subcortical contribute has been recently demonstrated (Ejaz et al., 2018). Intracellular recordings in animal stroke models revealed a hypoexcitability in the affected hemisphere and a hyperexcitability in the contralesional hemisphere (Buchkremer‐Ratzmann & Witte, 1997; Neumann‐Haefelin & Witte, 2000). In healthy humans, interhemispheric interactions have been studied in vivo by means of transcranial magnetic stimulation (TMS). In a pioneer study conducted by Ferbert et al., the authors used two TMS coils positioned over the two primary motor cortices (M1), demonstrating that a motor‐evoked potential (MEP) is inhibited by a pulse applied to the opposite M1 about 10–13 ms before (Ferbert et al., 1992). Since this mechanism is absent in patients with agenesis of the corpus callosum (CC), IHI is thought to reflect transcallosal transmission (Meyer, Röricht, Gräfin von Einsiedel, Kruggel, & Weindl, 1995).

In stroke patients, TMS studies investigating interhemispheric dynamics have given inconsistent results so far, with some works reporting no difference between the IHIs of the two hemispheres (Bütefisch, Wessling, Netz, Seitz, & Hömberg, 2008; Cassidy et al., 2015; Stinear, Petoe, & Byblow, 2015) and others reporting an imbalance between the affected (AH) and unaffected hemisphere (UH) (Borich, Neva, & Boyd, 2015; Dimyan et al., 2014). Thus, despite the large number of TMS studies conducted, interhemispheric dynamics in stroke populations are far from being fully elucidated. Different reasons can account for such variability. First, stroke often results in a disruption of corticospinal tract (CST) pathways, limiting or impeding, the evocation of an MEP even at high TMS intensity. Second, abnormalities in IHI may be more evident during tasks involving unimanual or bimanual movements, which can be performed only in a minority of well‐recovered stroke patients (Murase, Duque, Mazzocchio, & Cohen, 2004). Finally, it has to be considered that MEPs are not a direct index of cortical activity, being recorded by surface electromyography (EMG). For these reasons, there is the need of novel, reliable, and specific measures to test cortical dynamics in stroke patients.

In the present study, we used a novel approach by combining TMS and electroencephalography (EEG), which takes advantage of the strengths of the two techniques. TMS–EEG allows to focally stimulate a precise cortical area and, at the same time, to monitor neural activity both in the stimulated area and in the interconnected networks with an excellent temporal resolution and a good spatial resolution (Ilmoniemi et al., 1997). Recently, we introduced TMS–EEG as a valuable tool to investigate mechanisms of cortical reorganization early after stroke, even in the absence of recordable MEPs (Koch et al., 2019; Pellicciari et al., 2018). In another recent study of our group, we tested the sensitivity and reliability of two TMS–EEG indexes of interhemispheric interactions, that is, interhemispheric signal propagation (ISP) and interhemispheric balance (IHB) (Casula et al., 2020). Our results showed that these indexes have a low intersubject variability as well as a high test–retest reliability and, more importantly, showed a positive correlation with IHI, as measured with TMS–EMG. In the current study, our objective was to verify whether TMS–EEG measures of interhemispheric dynamics (i.e., ISP and IHB) are related to structural and clinical information of these patients, and thus potentially provide cortical markers of their neurophysiological state. In addition, we wanted to verify if these indexes were reliable, sensitive, and specific in unilateral stroke patients. To this aim, we applied TMS–EEG over M1 of the AH and the UH in a group of chronic stroke patients, which were compared with a group of age‐matched healthy controls (HC). Interhemispheric dynamics were evaluated in terms of signal propagation (ISP), balance (IHB), and connectivity (IHCoh). In addition, we evaluated whether TMS–EEG interhemispheric measures were linearly related to corticospinal, structural, and clinical data. In specific, to verify if signal propagation, as measured with ISP, was transcallosally mediated we tested if it was correlated with structural integrity of the CC, as measured with magnetic resonance imaging (MRI). To verify if ISP was related to excitatory and inhibitory circuits of the stimulated M1, we tested if it was correlated with corticospinal TMS–EMG measures (see methods section). In addition, to verify if ISP was predictive of the functionally recovery in unimanual movements, we tested correlation with an ad hoc test, namely the grip pinch force test (GPFT). Finally, to test the reliability of our measures, we repeated our evaluation after 3 weeks reporting data for each single subject.

## 2 METHODS

### 2.1 Patients and procedure

Then, 56 patients with a history of first‐ever unilateral ischemic stroke, admitted at the Santa Lucia Foundation for a standard rehabilitation, were screened for inclusion in this study. Also 19 patients (7 females; 61.26 ± 9.8 years) were enrolled in the study. Inclusion criteria were (a) first‐ever chronic ischemic stroke, that is, at least 6 months after the stroke event; (b) presence of hemiparesis due to an ischemic left or right subcortical or cortical lesion in the territory of the middle cerebral artery. Exclusion criteria were (a) history of seizures; (b) severe general impairment or concomitant disease; (c) patients older than 80 years; and (d) treatment with benzodiazepines, baclofen, and antidepressants. Table 1 summarizes the participants' demographic and clinical information. Each patient underwent two experimental sessions each comprising (a) a clinical evaluation; (b) a corticospinal evaluation, by means of TMS–EMG; (c) a cortical evaluation, by means of TMS–EEG; and (d) a structural evaluation, by means of an MRI scan (see below). The four evaluations were performed at baseline and after 3 weeks to test the reliability of our measures. Then, 14 right‐handed HC were recruited to be compared with the stroke patients in the cortical evaluation (8 females; 63.79 ± 12.87 years). The study was approved by the local ethics committee and written informed consent was obtained from each participant.

**TABLE 1:** Demographic and clinical information of the patients

*N*	Age	Gender	Stroke lesion	Affected hemisphere	Months from stroke	NIHSS	GPFT	RMT AH	RMT UH
**1**	**70**	**F**	**F–T, Ins**	**L**	**6**	**7**	**n.a.**	**66**	**74**
**2**	**74**	**F**	**CN, CR**	**R**	**6**	**9**	**n.a.**	**n.e.**	**75**
**3**	**61**	**F**	**F–P**	**L**	**13**	**7**	**−45.2**	**n.e.**	**54**
4	50	F	CN	R	7	7	−49.5	n.e.	65
5	79	F	Ins‐T–P–CN	R	10	6	n.a.	89	81.5
6	44	F	T‐ins	R	14	7	−43.7	n.e.	56
7	40	F	SC, CR	L	7	4	−4.7	63	72
**8**	**61**	**M**	**F–T–P**	**L**	**77**	**11**	**n.a.**	**n.e.**	**84**
9	58	M	F–T–P	R	5	2	−15.45	86	59
**10**	**64**	**M**	**F–P, Ins, CR, BG**	**L**	**12**	**8**	**−107.2**	**n.e.**	**56**
**11**	**54**	**M**	**F–T–P, LN, IC**	**R**	**13**	**4**	**−23.3**	**60**	**49**
**12**	**64**	**M**	**CR, EC**	**L**	**6**	**8**	**−25**	**60**	**57.5**
13	59	M	F–T–P	R	7	10	−63.3	n.e.	56
**14**	**67**	**M**	**F–T–P**	**R**	**24**	**5**	**n.a.**	**71.5**	**58**
15	62	M	F‐Ins, P–O, PUT	R	6	10	n.a.	n.e.	86
**16**	**62**	**M**	**F–T–P**	**L**	**7**	**4**	**n.a.**	**50**	**46**
17	70	M	F–P	R	78	3	−68.6	87	57
18	55	M	CN	R	6	4	n.a.	96	75
**19**	**70**	**M**	**F–P**	**R**	**16**	**10**	**n.a.**	**n.e.**	**71.5**

*Note*: Instrumental and neurophysiological data are averaged between the two time points (T0 and T1). RMT values refer to the ones collected in the cortical evaluation. Patients who underwent structural evaluation are highlighted in bold.

Abbreviations: AH, affected hemisphere; BG, basal ganglia; CN, capsular nucleus; CR, corona radiata; EC, external capsula; F, female; F–T–P–O, fronto–temporal–parietal–occipital; IC, internal capsula; Ins, Insula; LN, lenticular nucleus; M, male; mRS: modified Rankin Scale; NIHSS: National Institute of Health Stroke Scale; PUT, putamen; R, real‐TBS; RMT, resting motor threshold; S, sham‐TBS; SC, semioval center; UH, unaffected hemisphere.

### 2.2 Clinical evaluation

Clinical evaluation was made using the National Institutes of Health Stroke Scale (NIHSS) and the GPFT. The latter was performed with a digital hand‐held dynamometer (Center for Innovative Technics BY; The Netherlands). The patient was placed seated with the elbows resting on the table top, with a 90° flexion of elbow and neutral forearm (Richards & Olney, 1996). Four hand grips at maximal force were tested with suitable hand pieces (Stock, Thrane, Askim, Anke, & Mork, 2019): (a) whole hand power grip; (b) pincer grasp, that is, opposition between thumb and index finger; (c) key grip, that is, volar side of index and thumb finger; (d) thumb opposition to volar sides of middle and ring finger. Each trial was repeated three times, starting from the nonaffected side. The hemiplegic arm was tested immediately after the nonaffected arm. For each grip the average (in Newton) of the three repetitions was calculated, the results of the test were compared between affected and nonaffected side generating an indicative result of the difference in strength.

### 2.3 Corticospinal evaluation

Analysis of corticospinal activity was performed with TMS–EMG. Single‐pulse TMS was carried out using a Magstim 200 stimulator with a 70 mm figure‐of‐eight coil (Magstim Company Limited, Whitland, UK), which produces a monophasic pulse of ∼80 μs length. The position of the coil on the scalp was defined as the M1 site in which TMS evoked the largest MEPs in the relaxed first dorsal interosseous (FDI) muscle of the hand contralateral to the stimulation. The coil was oriented tangentially to the scalp at about 45° angle away from the midline, thus inducing a posterior–anterior current in the brain. The intensity of stimulation for single‐pulse TMS was adjusted to evoke a 1 Mv MEP. To this aim, we first tested the resting motor threshold (RMT), defined as the lowest intensity that produced MEPs >50 μV in at least 5 out of 10 trials in the relaxed FDI of the right hand (Rossini et al., 1994). Then, starting from 130% of RMT, we looked for an intensity able to evoke an MEP with, on average of 15 trials, a peak‐to‐peak amplitude of about 1 mV. Paired‐pulse TMS was carried out with two Magstim 200 stimulators connected by a Bistim module and two 70 mm figure‐of‐eight coils. Intensity of paired‐pulse TMS was based on the RMT or on the active motor threshold (AMT), defined as the lowest intensity that produced MEPs >200 μV in at least 5 out of 10 trials during 10% of maximum contraction of the same muscle (Rothwell, 1997). Paired‐pulse TMS consisted in (a) short‐interval cortical inhibition and intracortical facilitation (SICI/ICF), in which a conditioning stimulus (CS) delivered at 90% of AMT preceded a test stimulus (TS) delivered at 1 mV MEP intensity over M1 by 1, 2, 3, 5, 7, 10, and 15 ms (Kujirai et al., 1993; Rocchi et al., 2017); (b) long‐interval cortical inhibition (LICI), in which a CS delivered at 100% of RMT preceded a TS delivered at 1 mV MEP intensity over M1 by 50, 100, and 150 ms. Ten TMS paired pulses were delivered for each ISI (Valls‐Solé, Pascual‐Leone, Wassermann, & Hallett, 1992); and (c) IHI, in which a CS delivered at 1 mV MEP intensity over one M1 preceded a TS delivered at 1 mV MEP intensity over the contralateral M1 by 10 ms. Ten TMS paired pulses were delivered for each M1 (Ferbert et al., 1992).

SICI/ICF, LICI, and IHI were assessed over the UH‐M1 and, when MEP was evocable, over the AH‐M1. Corticospinal excitability was assessed by peak‐to‐peak MEP amplitude. To measure MEPs, EMG was recorded from the FDI muscle contralateral to the stimulation using 9‐mm‐diameter Ag–AgCl surface cup electrodes. The active electrode was placed over the belly muscle, whereas the reference electrode was located over the metacarpophalangeal joint of the index finger. Responses were amplified using a Digitimer D360 amplifier through filters set at 5 Hz and 2 kHz with a sampling rate of 5 kHz and then recorded by a computer using SIGNAL software (Cambridge Electronic Devices).

### 2.4 Cortical evaluation

Analysis of cortical activity was performed with TMS–EEG. TMS was carried out using a Magstim R^2^ stimulator with a 50 mm figure‐of‐eight coil (Magstim Company Limited), which produces a biphasic waveform with a pulse width of ∼0.1 ms. Coil positioning was the same used for corticospinal evaluation. Intensity of stimulation was set at 90% of the RMT. When RMT was not recordable in the AH due to lack of MEP response, TMS was set at the same value of the UH. Each session consisted of 80 TMS pulses applied at a random ISI of 2–4 s over M1 of both the hemispheres separately, that is, the UH and the AH. The order of stimulation of the two hemispheres was counterbalanced across patients. During the entire session, patients were seated on a dedicated, comfortable armchair in a soundproofed room. A TMS‐compatible DC amplifier (BrainAmp, Brain Products GmbH, Munich, Germany) was used to record EEG activity from the scalp. The EEG was continuously recorded from 29 scalp sites positioned according to the 10–20 International System, using TMS‐compatible Ag/AgCl pellet electrodes mounted on an elastic cap. Additional electrodes were used as ground and reference. The ground electrode was positioned in AFz, while the reference was positioned on the tip of the nose. EEG signals were digitized at a sampling rate of 5 kHz. Skin/electrode impedance was maintained below 5 kΩ. Horizontal and vertical eye movements were detected by recording the electrooculogram to offline reject the trials with ocular artifacts.

TMS–EEG data were analyzed offline with Brain Vision Analyzer (Brain Products GmbH) and EEGLAB toolbox running in a MATLAB environment (MathWorks Inc., Natick, MA). As a first step, data were segmented into epochs starting 1 s before the TMS pulse and ending 1 s after it. We first removed and then replaced data, using a cubic interpolation, from 1 ms before to 10 ms after the TMS pulse from each trial. Afterward, data were downsampled to 1,000 Hz and band‐pass filtered between 1 and 80 Hz (Butterworth zero phase filters). A 50 Hz notch filter was applied to reduce noise from electrical sources. Then, all the epochs were visually inspected and those with excessively noisy EEG were excluded from the analysis. Independent component analysis (INFOMAX‐ICA) was applied to the EEG signal to identify and remove components reflecting muscle activity, eye movements, blink‐related activity, and residual TMS‐related artifacts basing on previously established criteria (Casula et al., 2017). Finally, the signal was rereferenced to the average signal of all the electrodes. For descriptive purposes, we collapsed data from the stimulation of the AH on the left hemisphere (LH), whereas data from UH stimulation were collapsed over the right one (RH), the same procedure was done for MRI lesion overlapping.

TMS‐evoked activity was analyzed in the temporal, spatial, and oscillatory domain. First, we rectified the TMS‐evoked activity recorded over three electrodes surrounding the two M1s, that is, C3, CP3, CP5 for the left M1 and C4, CP4, CP6 for the right M1. These electrodes were chosen basing on previous TMS–EEG studies assessing M1 local excitability (e.g., (Casula et al., 2016; Jarczok et al., 2016; Määttä et al., 2017)). We then averaged the amplitude of the rectified TMS‐evoked activity from 20 to 150 ms after the TMS pulse for the stimulated M1 and from 30 to 160 ms for the M1 contralateral to the stimulation. These time windows were chosen based on the (a) mean duration of the GABA‐receptor‐mediated inhibitory neurotransmission, that is, ≈150 ms (Fitzgerald, Maller, Hoy, Farzan, & Daskalakis, 2009; Jarczok et al., 2016; Määttä et al., 2017; Voineskos et al., 2010) and (b) transcallosal interhemispheric latency, that is, ~10 ms (Ferbert et al., 1992; Jarczok et al., 2016). Finally, we computed the ISP with the following formula:

$$\mathrm{ISP}=\frac{\mathrm{TMS}\;\text{evoked activity}\;\left(\text{non stimulated}\;M1\right)}{\mathrm{TMS}\;\text{evoked activity}\;\left(\text{stimulated}\;M1\right)}$$

To assess the balance between the two hemispheres, we computed the IHB as follows:

$$\mathrm{IHB}=\frac{{\mathrm{ISP}}_{\mathrm{AH}}}{{\mathrm{ISP}}_{\mathrm{UH}}}$$

where ISP_AH_ and ISP_UH_ are the ISP computed after the stimulation of M1 in the AH and AH, respectively (Casula et al., 2020). To assess the interhemispheric connectivity, we computed spectral coherence between the two M1 clusters of electrodes considering two epochs: a reference period, from 500 ms before to 20 ms before each TMS pulse, and an interest period, from 20 to 500 ms after each TMS pulse. For each subject and condition, we computed the power spectra for each single epoch and frequency between 4 and 40 Hz, by means of a fast Fourier transform (Hamming window; frequency resolution 1.5 Hz). Coherence values for all frequency bins were computed with the following formula:

$$\text{IHCoh}\left(f\right)=\frac{{\left|\mathrm{CS}\left(c_{1},c_{2}\right)\left(f\right)\right|}^{2}}{\left(\left|\mathrm{CS}\left(c_{1},c_{2}\right)\left(f\right)\right|\left|\mathrm{CS}\left(c_{1},c_{2}\right)\left(f\right)\right|\right)}$$

With *CS* = (*c*_1_, *c*_2_)(*f*) = *sigma*; (*c*_1_, *i*(*f*)(*c*_2_, *i*(*f*), where *i* represents the epoch number. This formula extends the Pearson's correlation coefficient to complex number pairs. Accordingly, the coherence spectrum of two signals (*c*_1_ and *c*_2_) is computed as normalization of cross‐spectrum by the two auto‐spectra. For each frequency *f*, the coherence value is a real number between 0 and 1. Coherence values were then obtained by averaging the values over all the epochs for the alpha band (8–13 Hz), which was found as the natural frequency of M1, as revealed by a time/frequency decomposition based on a complex Morlet wavelet transform (cycles = 3.5). Finally, event‐related coherence *ERC*_*C*1,*C*2_ was obtained by subtracting the reference period value (COH_*C*1,*C*2_ reference) from the corresponding interest period value (COH_*C*1,*C*2_ interest), according to the following formula:

$${\mathrm{ERC}}_{C1,C2}={\mathrm{Coh}}_{C1,C2}\;\text{interest}-{\mathrm{Coh}}_{C1,C2}\;\text{reference}$$

Therefore, a coherence increase in the frequency band during COH_*C*1,*C*2_ interest relative to COH_*C*1,*C*2_ reference is expressed as a positive value, while a coherence decrease is expressed by a negative value (Fuggetta, Pavone, Fiaschi, & Manganotti, 2008; Pfurtscheller & Lopes da Silva, 1999). IHCoh was computed both for the AH (IHCoh_AH_) and for the UH (IHCoh_UH_).

### 2.5 Structural evaluation

Structural brain MRI was obtained in a single session using a head‐only 3.0 T scanner (Siemens Magnetom Allegra, Siemens Medical Solution, Erlangen, Germany), equipped with a circularly polarized transmit‐receive coil. The acquisition protocol included the following sequences: (a) dual‐echo turbo spin echo (repetition time [TR] = 8,770 ms; echo time [TE] = 12/109 ms); (b) FLAIR (TR = 9,350 ms, TE = 60 ms); (b) morphological 3D‐T1‐weighted magnetization‐prepared rapid acquisition gradient echo (MPRAGE) (TR = 2,500 ms; TE = 2.74 ms; inversion time = 900 ms; flip angle = 8°; matrix = 256 × 208 × 176; FoV = 256 × 208 × 176 mm^3^); (d) diffusion weighted spin‐echo echo planar imaging (twice‐refocused SE EPI) (TR = 170 ms, TE = 85 ms, maximum b factor = 1,000 s mm^−2^, isotropic resolution = 2.3 mm^3^), collecting seven images with no diffusion weighting (b0) and 61 images with diffusion gradients applied in 61 non collinear directions (scan time: 11 min).

Diffusion data were processed using tools from the FMRIB software library (FSL, www.fmrib.ox.ac.uk/fsl/) and from CAMINO (www.camino.org.uk). After correction for eddy current induced distortions and involuntary motion (performed using eddy correct, available with FSL), the diffusion tensor was estimated voxel‐wise (Basser, Mattiello, & LeBihan, 1994) using CAMINO. Then, a map of fractional anisotropy (FA) and mean diffusivity (MD) was obtained computed for every subject (Cook et al., 2006). The CC was reconstructed in native space for every subject with multifiber probabilistic tractography, carried out using 10,000 iterations of the probabilistic index of connectivity algorithm (Parker, Haroon, & Wheeler‐Kingshott, 2003) applied to fiber orientation distribution functions estimated with persistent angular structure PAS MRI (Jansons & Alexander, 2003). Five principal parts of the CC were reconstructed separately, using as seeds the regions resulting from parcellation of the midsagittal section (Hofer & Frahm, 2006): genu (Region I), anterior midbody (Region II), posterior midbody (Region III), isthmus (Region IV), and splenium (Region V). The FA and MD of each portion was estimated and used for the correlation analysis described in the results section (Makovac et al., 2016).

For each patient, lesions were outlined in the MPRAGE images, using a semi‐automated local threshold contouring software (Jim 5.0, Xinapse System, Leicester, UK, http://www.xinapse.com/). A lesion mask was created for each patient by assigning a value of 1 to every voxel corresponding to a lesion and a value of 0 elsewhere. T1‐weighted images were warped into the Montreal Neurological Institute space. The same transformation was applied to the corresponding lesion mask. A probabilistic lesion map, indicating the percentage of patients with a lesion in a given area, in the LH, was obtained by combining every patient's lesion masks.

### 2.6 Statistical analysis

All data were analyzed using SPSS version 22 (SPSS Inc., Chicago, IL). Prior to undergoing analysis of variance (ANOVA) procedures, normal distribution of clinical and neurophysiological data was assessed by means of Shapiro–Wilks' test. Level of significance was set at *α* = .05. Sphericity of the data was tested with Mauchly's test; when sphericity was violated (i.e., Mauchly's test <0.05) the Huynh–Feldt *ε* correction was used. Pairwise comparisons were corrected by the Bonferroni method.

GPFT scores in the two sessions were compared with Wilcoxon sum rank test, since they were not normally distributed. ICF/SICI, LICI, and IHI were analyzed by means of a one‐way ANOVA with a within‐subject factor “ISI.” This analysis was performed only for the UH since we were not able to test these measures from the AH (see Section 3). RMT and IHB were analyzed by means of a one‐way ANOVA with a between‐subjects factor “group” (stroke vs. HC). TMS‐evoked activity was first analyzed by means of a two‐way mixed ANOVA with between‐subjects factor “group” (stroke patients vs. HC) and “stimulation” (LH/AH vs. RH/UH) to test possible difference in local cortical activation of the only stimulated hemisphere. Then, to assess differences in the interhemispheric dynamics between the two groups, TMS‐evoked cortical activity was analyzed by means of a four‐way mixed ANOVA with between‐subjects factor “group” and “MEP” (evocable vs. not‐evocable) and within‐subject factors “stimulation” (LH/AH vs. RH/UH) and “hemisphere” (stimulated vs. contralateral). The MEP factor was added to test if the presence of a MEP from the AH could have an effect on our results. To further test this possibility, we repeated the same analysis including only the stroke patients with a clearly evocable MEP from the AH (see results). ISP and IHCoh were analyzed by means of a two‐way mixed ANOVA with factor “group” and “stimulation.” Test–retest reliability of ISP, IHB, and IHCoh was assessed by means of standard error of measurement (SEM_eas_) and small detectable change (SDC) (Schambra et al., 2015). SEM_eas_ was computed as the standard deviation of all within‐subject sources of variance, in our case, the two sessions, without considering the between‐subjects variance:

$${\text{SEM}}_{\text{eas}}=\sqrt{\sigma_{\text{session}}^{2}+\sigma_{\text{residual}}^{2}}$$

SEM_eas_ was then used to compute SDC_indiv_, which is the smallest change in a measurement that can be considered a real change above measurement noise (Beckerman et al., 2001; Schambra et al., 2015). SDC_indiv_ was computed as:

$${\mathrm{SDC}}_{\text{indiv}}={\mathrm{SEM}}_{\mathrm{eas}}*\sqrt{2}*1.96$$

where $\sqrt{2}$ accounts for the variances associated with the two experimental sessions and 1.96 defines the 95% confidence interval. We then computed the SDC for a group (SDC_group_) for an *n* sample size as:

$${\mathrm{SDC}}_{\text{group}}=\frac{{\mathrm{SDC}}_{\text{indiv}}}{\sqrt{n}}$$

To assess whether test–retest reliability of these measures was different in stroke patients as compared with healthy volunteers, we compared SDC of the sample of patients with a sample of 20 healthy volunteers tested in a previous study of our group (Casula et al., 2020) with unpaired *t*‐test. To assess the sensitivity of ISP, IHB and IHCoh in distinguishing the group of healthy volunteers from stroke patients, we used intraclass correlation coefficient (ICC), which indicates how strongly units in the same group resemble each other. For the ICC calculation, we considered all the between‐subjects sources of variance and we computed it as:

$$\mathrm{ICC}=\frac{\sigma_{\text{subjects}}^{2}}{\sigma_{\text{subjects}}^{2}+\sigma_{\text{sessions}}^{2}+\sigma_{\text{residual}}^{2}}$$

ICC range from 0 to 1, we established a cut‐off of 0.7 indicating a relatively stable and reliable measure to distinguish between the two groups (Brown et al., 2017; Schambra et al., 2015; Shrout & Fleiss, 1979).

In order to explore linear relationships between clinical, structural, and neurophysiological outcomes of stroke patients collected in the two experimental sessions, we tested correlations using the Pearson's coefficient or the nonparametric Spearman's coefficient, when data distribution was not normal. In specific, we tested linear relationships between GPFT score and IHB, given that both measures are derived from a balance between the strength of the two upper limbs (GPFT) and of the two‐hemisphere activity (IHB). Moreover, we explore linear relationships between ISP and IHCoh from the AH and UH stimulation with (a) FA of the CST afferent from the AH and UH and (b) FA of the splenium (Region V of the CC) given its specific role of this structure in the interhemispheric connection of motor (Wahl et al., 2007) and nonmotor areas (Koch et al., 2011). Given the exploratory nature of our correlation analysis, we did not corrected our *p*‐values for multiple correlations.

## 3 RESULTS

The entire procedure was well tolerated, and no significant side effects were reported. Results of the clinical evaluation are reported in Table 1.

### 3.1 Clinical evaluation

All the 19 patients were successfully screened with NIHSS (mean score 6.63 ± 0.80). Six patients showed a score between 1 and 4 (mean score 3.5 ± 0.34), indicating a “minor stroke” severity; the other 13 patients showed a score between 5 and 11 (8.07 ± 0.45) indicating a “moderate stroke” severity. Eight patients were excluded from the evaluation with the GPFT due to excessive spasticity of the hand (MAS > 2). Analysis of the GPFT did not reveal any significant difference between the two experimental sessions (first: −46.87 ± 8.84, second: −42.96 ± 8.46; *p* = .06).

### 3.2 Corticospinal evaluation

Twelve patients did not have a sufficiently stable 1 mV MEP to test IHI, ICF/SICI, and LICI in the AH. Of these, nine patients did not show any evocable MEP; the other three patients showed sufficiently stable MEPs of about 50 μV thus we could assess RMT, although at a very high intensity value, respectively, 96, 89, and 87% of MSO (Table 1). Thus, the analysis was conducted only for the UH for ICF/SICI and LICI protocols. RMT did not differ between the two groups for the AH/LH (stroke: 65.42 ± 4.27, HC: 68 ± 3.02; *p* = .67) nor for the UH/RH (stroke: 69.16 ± 2.76, HC: 67.14 ± 3.13; *p* = .66). Analysis of the UH‐SICI showed a significant effect of ISI (*F*(6,90) = 14.355; *p* < .001; *ε* = .489). Post hoc analysis showed a lower corticospinal excitability at an ISI of 1 and 2 ms compared to ISIs at 5, 7, 10, and 15 ms (all *p*s < .01); a significant difference was observed also between the ISI between 3 and 7 ms (*p* = .003). Analysis of the UH‐LICI did not show any significant main effect nor interactions.

### 3.3 Cortical evaluation

**FIGURE 1:**
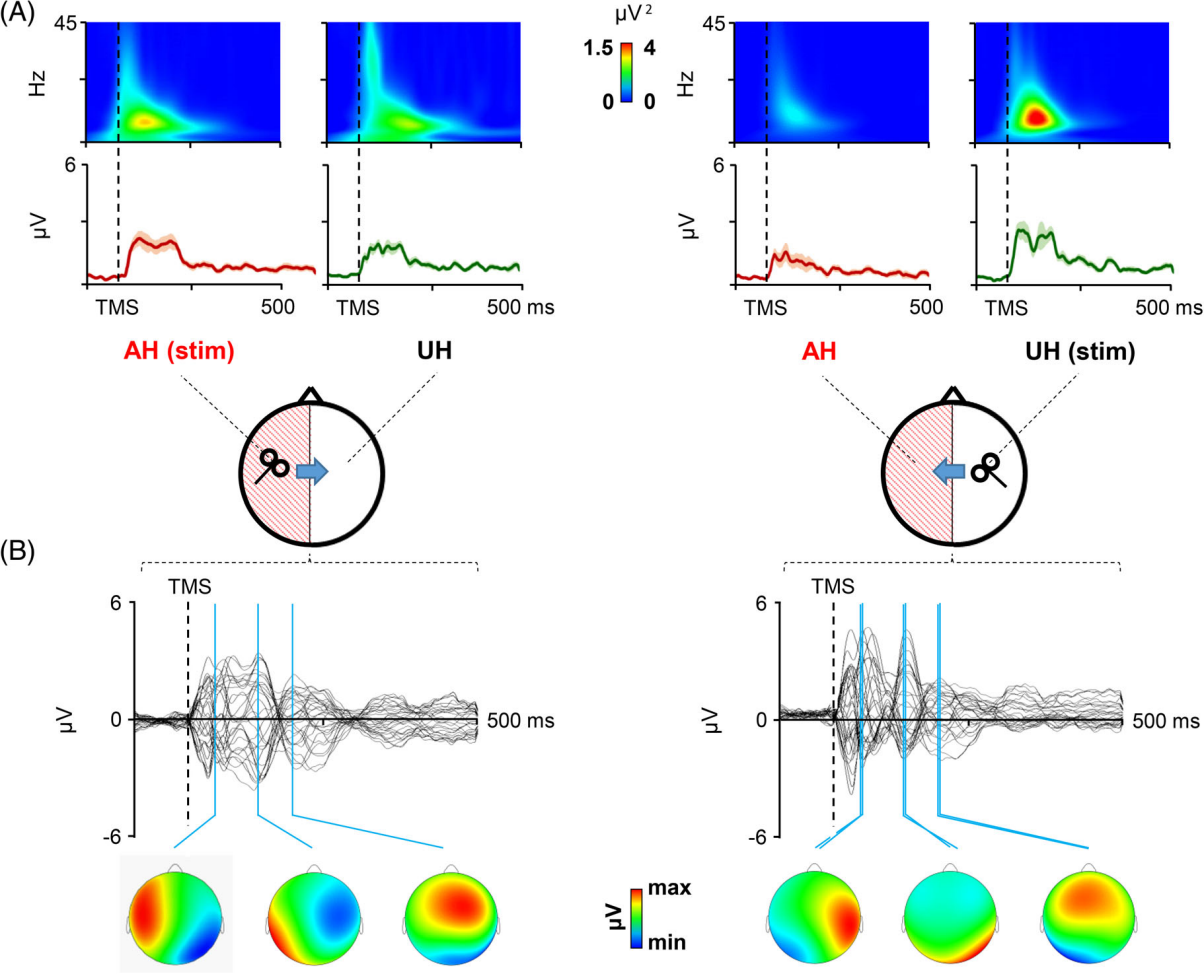
Local and global transcranial magnetic stimulation (TMS)‐evoked cortical response after stimulation of the affected hemisphere (AH) and unaffected hemisphere (UH) in stroke patients. Panel (a): Local cortical response is displayed in terms of TMS‐evoked activity and cortical oscillations evoked over primary motor cortices (M1). Panel (b): Global cortical response is displayed in terms of TMS‐evoked potentials (TEPs) recorded over all the scalp with the scalp voltage distribution at the three main peaks of activity (20–40 ms, 40–70 ms, and 70–150 ms)

**FIGURE 2:**
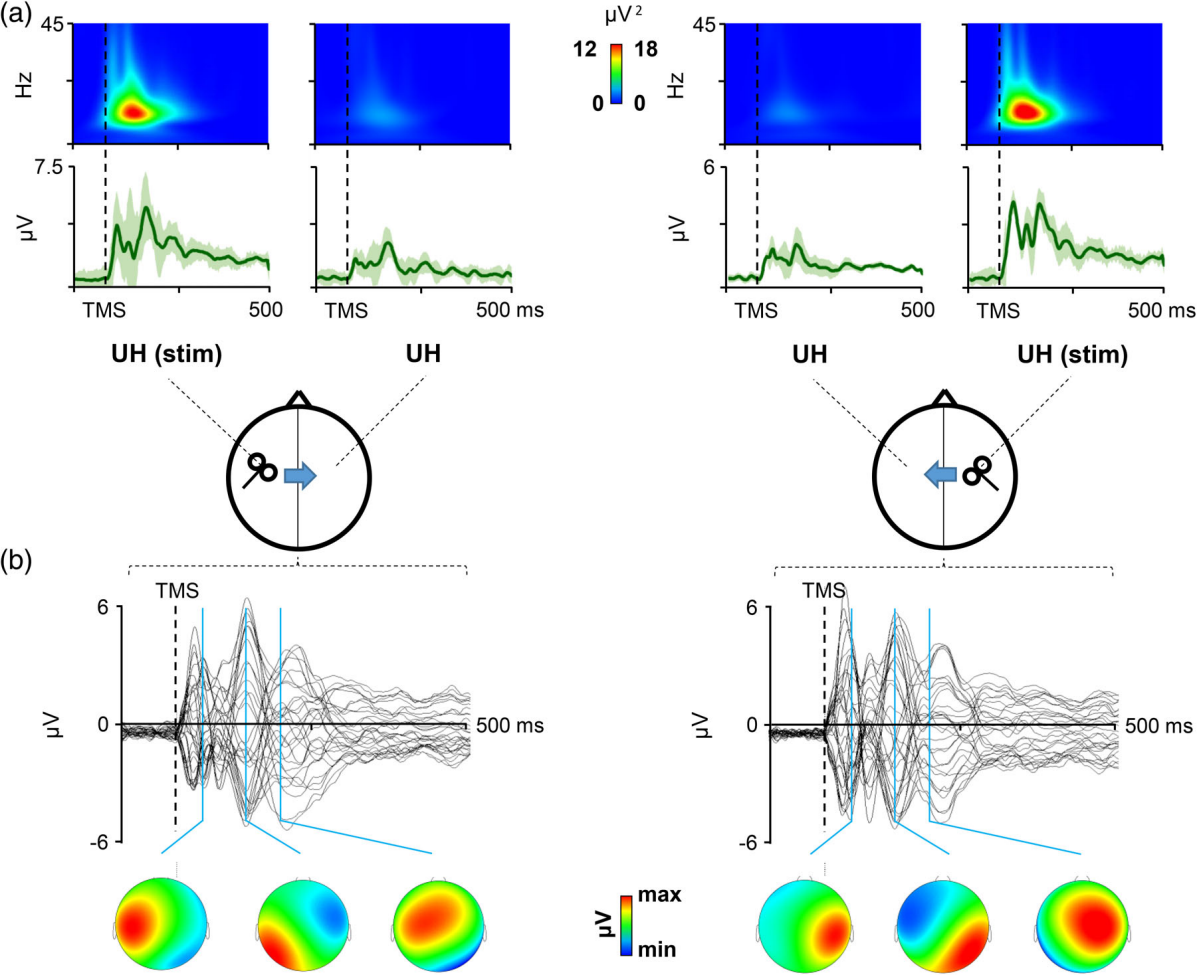
Local and global transcranial magnetic stimulation (TMS)‐evoked cortical response after stimulation of the two hemispheres in healthy controls (HC). Panel (a): Local cortical response is displayed in terms of TMS‐evoked activity and cortical oscillations evoked over M1. Panel (b): Global cortical response is displayed in terms of TMS‐evoked potentials (TEPs) recorded over all the scalp with the scalp voltage distribution at the three main peaks of activity (20–40 ms, 40–70 ms, and 70–150 ms)

All the 19 patients were able to complete the cortical evaluation. Figures 1 and 2 depict the local and global cortical response following stimulation of M1 in stroke patients (Figure 1) and HC (Figure 2). Temporal analysis of local M1 TMS‐evoked activity revealed a sustained cortical response lasting ≈250 ms, with a maximum activation at ≈100–150 ms; the same temporal dynamic was revealed by the time‐frequency analysis showing a maximum activation at ≈100–150 ms in the alpha frequency. Pattern of activation was similar, in terms of waveform and amplitude, between the stimulations of two hemispheres in HC, with a strong reduction of activity in the hemisphere contralateral to the stimulation (Figure 2a). In stroke patients, interhemispheric reduction of TMS‐evoked activity was observable only when stimulating the UH, but not when stimulating the AH (Figure 1a). Spatiotemporal reconstruction of global TMS‐evoked cortical activity (Figure 1b stroke patients; Figure 2b HC) revealed a well‐known sequence of positive and negative deflections lasting ≈250 ms, as usually observed after M1 stimulation (Casula et al., 2016; Casula, Mayer, et al., 2018; Casula, Rocchi, Hannah, & Rothwell, 2018). A first activation was focused over the stimulated M1 (20–40 ms) with an immediate spread over ipsilateral posterior areas (40–70 ms) and frontal areas (100 ms). At 150 ms, we observed a prominent bilateral distribution over both the hemispheres. This pattern was observable in both stroke and HC and occurred in a similar way in the two hemispheres.

**FIGURE 3:**
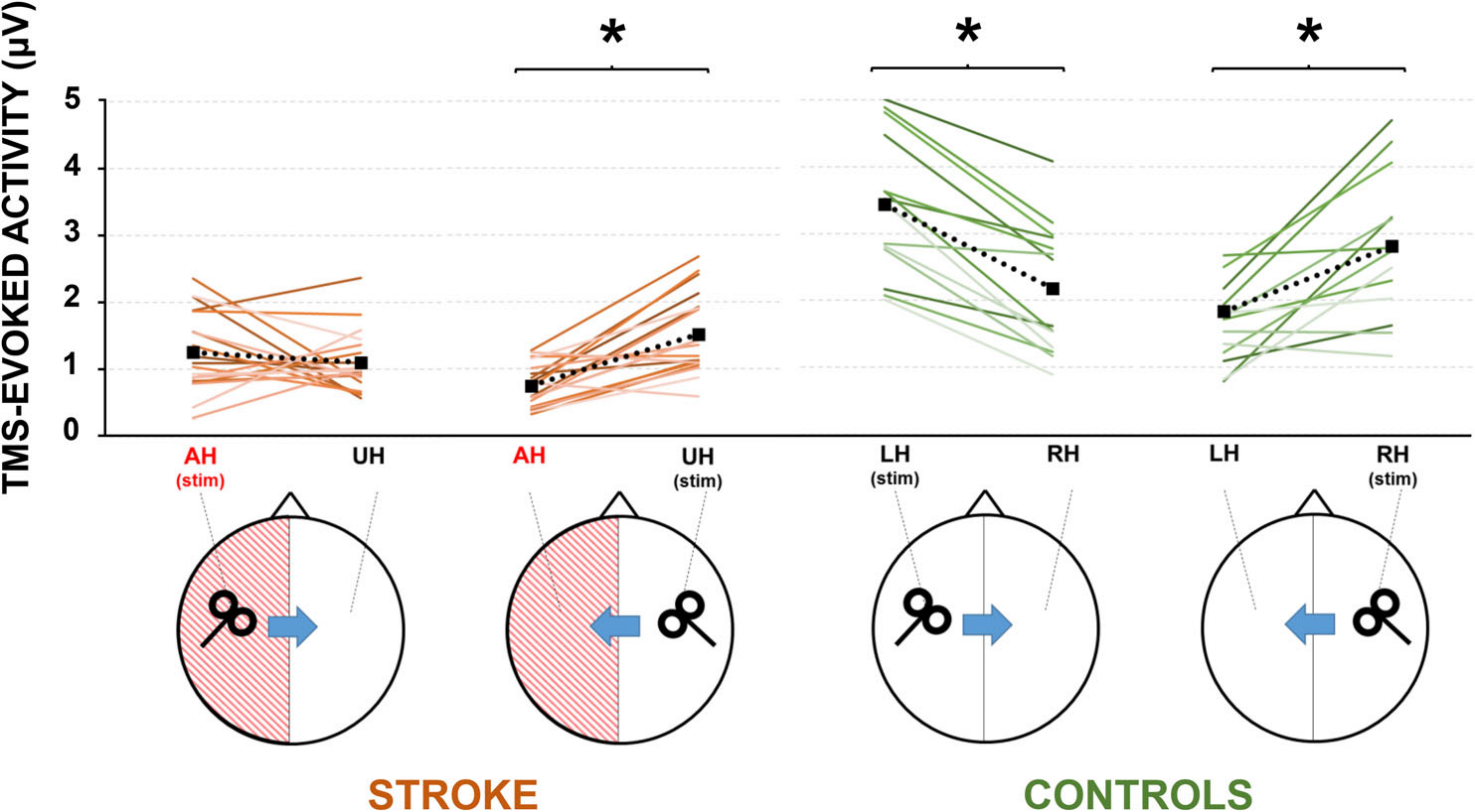
Analysis of local transcranial magnetic stimulation (TMS)‐evoked cortical activity evoked from affected (AH) and unaffected (UH) M1 in stroke patients (brown lines) and left and right M1 in healthy controls (green lines). The plots depict the amplitude of the TMS‐evoked cortical activity evoked in the stimulated hemisphere and in the contralateral one for each single subject

Figure 3 depicts the analysis of local TMS‐evoked cortical activity in the two groups. The first analysis aimed at testing differences between the cortical activation of the two hemispheres when directly stimulated, revealed a group × stimulation interaction (*F*(1,31) = 5.855; *p* = .022; *ε* = .159). Post hoc analysis revealed no differences between the activation of the two hemispheres in stroke patients (*p* = .073) nor in HC (*p* = .131). The second analysis aimed at testing differences in the interhemispheric dynamics revealed a group × stimulation × hemisphere interaction (*F*(1,31) = 14.009; *p* = .001; *ε* = .318). No main effect was observable of the MEP factor (*p* = .947) nor in interaction with other factors (all *p*s > .2). Post hoc analysis comparing the two groups revealed a lower excitability in stroke patients, compared to HC, both when stimulating the AH (1.25 ± 0.59 μV vs. 2.79 ± 1.25 μV; *p* < .001) and when stimulating the UH (1.51 ± 0.59 μV vs. 2.32 ± 1.09 μV; *p* = .027). Post hoc analysis comparing the two hemispheres reactivity in HC, revealed a higher excitability over the stimulated hemisphere, compared to the contralateral one, as expected (LH stimulation: 2.79 ± 1.25 vs. 1.18 ± 0.40 μV, *p* < .001; RH stimulation: 2.32 ± 1.09 vs. 1.06 ± 0.41 μV; *p* < .001). In stroke patients, this difference was significant only when stimulating the UH (1.51 ± 0.59 vs. 0.74 ± 0.30 μV; *p* = .001) but not when stimulating the AH (1.25 ± 1.09 vs. 1.10 ± 0.50 μV; *p* = .39). When we considered only the patients with a clearly MEP evocable from the AH, the analysis confirmed the significant group × stimulation × hemisphere interaction (*F*(1,22) = 14.525; *p* = .001; *ε* = .398). Post hoc analysis confirmed that TMS‐evoked cortical activity was significantly inhibited stimulating both the hemispheres of HC (all *p*s < .001) and when stimulating the UH of stroke patients with AH‐RMT (*p* = .027) but not when stimulating the AH (*p* = .945).

**FIGURE 4:**
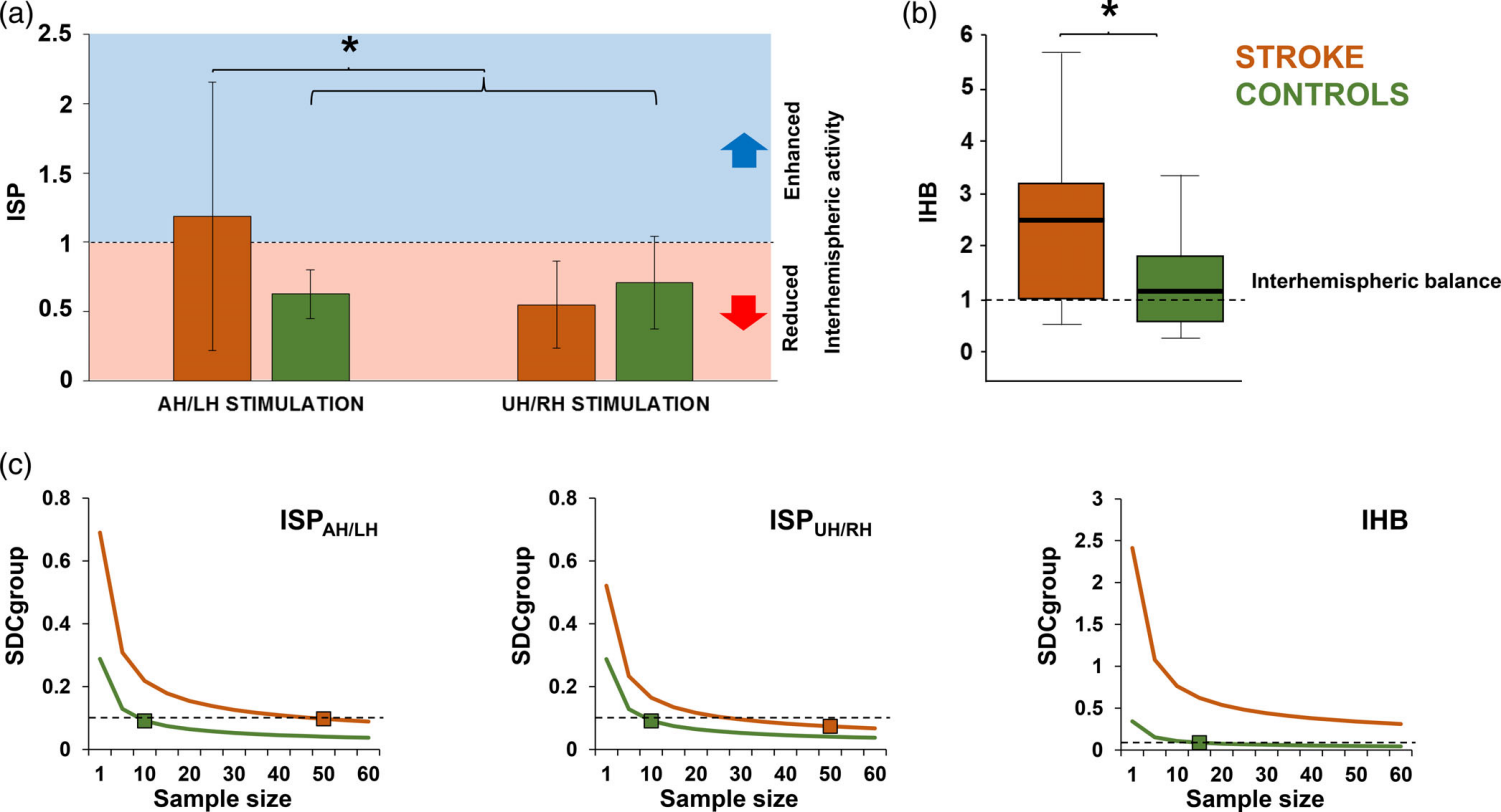
Analysis of interhemispheric signal propagation (ISP) and interhemispheric balance (IHB). Panel (a): ISP amplitude after stimulation of left/affected hemisphere and right/unaffected hemisphere for stroke patients (brown bars) and healthy controls (green bars). Panel (b): IHB for stroke patients (brown bar) and healthy controls (green bar). IHB ≃ 1 indicates IHB (dotted line). Panel (c): Small detectable change at group level (SDC_group_) for ISP and IHB computed for an *n* sample size (1–60) of stroke patients. Brown and green squares indicate the sample size necessary to obtain an SDC_group_ of less than 0.1 (dotted line)

Figure 4a depicts the analysis of ISP. This analysis revealed a group × stimulation interaction (*F*(1,31) = 8.242; *p* = .007; *ε* = .210). Post hoc analysis comparing the two groups, revealed a higher ISP (i.e., lower IHI) when stimulating the AH in stroke patients compared to HC (1.18 ± 0.96 vs. 0.49 ± 0.26; *p* = .014); while no difference was observable when stimulating the UH (0.55 ± 0.22 vs. 0.59 ± 0.38; *p* = .703). When comparing the two stimulated hemispheres, stroke patients showed a higher ISP (i.e., lower IHI) when stimulating the AH compared to the UH (*p* = .001), whereas, as expected, HC did not show any significant difference between the two stimulations (*p* = .6).

Figure 4b depicts the analysis of IHB. This analysis revealed a main effect of group (*F*(1,31) = 5.12; *p* = .015; *ε* = .142). Post hoc analysis revealed that IHB was significantly higher in stroke patients compared to HC (2.53 ± 2.45 vs. 1.18 ± 0.87). Analysis on IHCoh did not reveal any main effect of group or stimulus factors, nor any interaction. Analysis of test–retest reliability for stroke patients revealed the following SEM_eas_ for ISP_AH_ (0.249), ISP_UH_ (0.187), IHB (0.87), IHCoh_AH_ (0.065), and IHCoh_UH_ (0.099) with the following SDC_indiv_ necessary to exceed measurement noise for ISP_AH_ (0.691), ISP_UH_ (0.521), IHB (2.41), IHCoh_AH_ (0.181), and IHCoh_UH_ (0.274). Analysis of test–retest reliability for healthy volunteers revealed the following SDC_indiv_ necessary to exceed measurement noise for ISP_AH_ (0.288), ISP_UH_ (0.287), and IHB (0.343) (we excluded IHCoh from this analysis since the ICC for this measure was not reliable, see below). Figure 4c depicts analysis of SDC_group_ for both groups. To reduce SDC_group_ below 0.1 in stroke patients the following sample sizes would be needed: 50 for ISP_AH_, 35 for ISP_UH_ and more than 60 for IHB. The same analysis conducted on healthy volunteers revealed that 10 subjects would be needed for ISP regardless the hemisphere of stimulation and 15 for IHB. Analysis of SDC comparison showed that a higher reliability of these measures in healthy volunteers compared to stroke patients (all *p*s < .05). Analysis of ICC revealed a high reliability in distinguish between the two groups for IHB (0.838; *p* < .001), ISP_AH_ (0.888; *p* < .001) and ISP_UH_ (0.865; *p* < .001), but not for IHCoh_AH_ (0.315; *p* = .215) nor for IHCoh_UH_ (0.483; *p* = .09).

### 3.4 Structural evaluation

**FIGURE 5:**
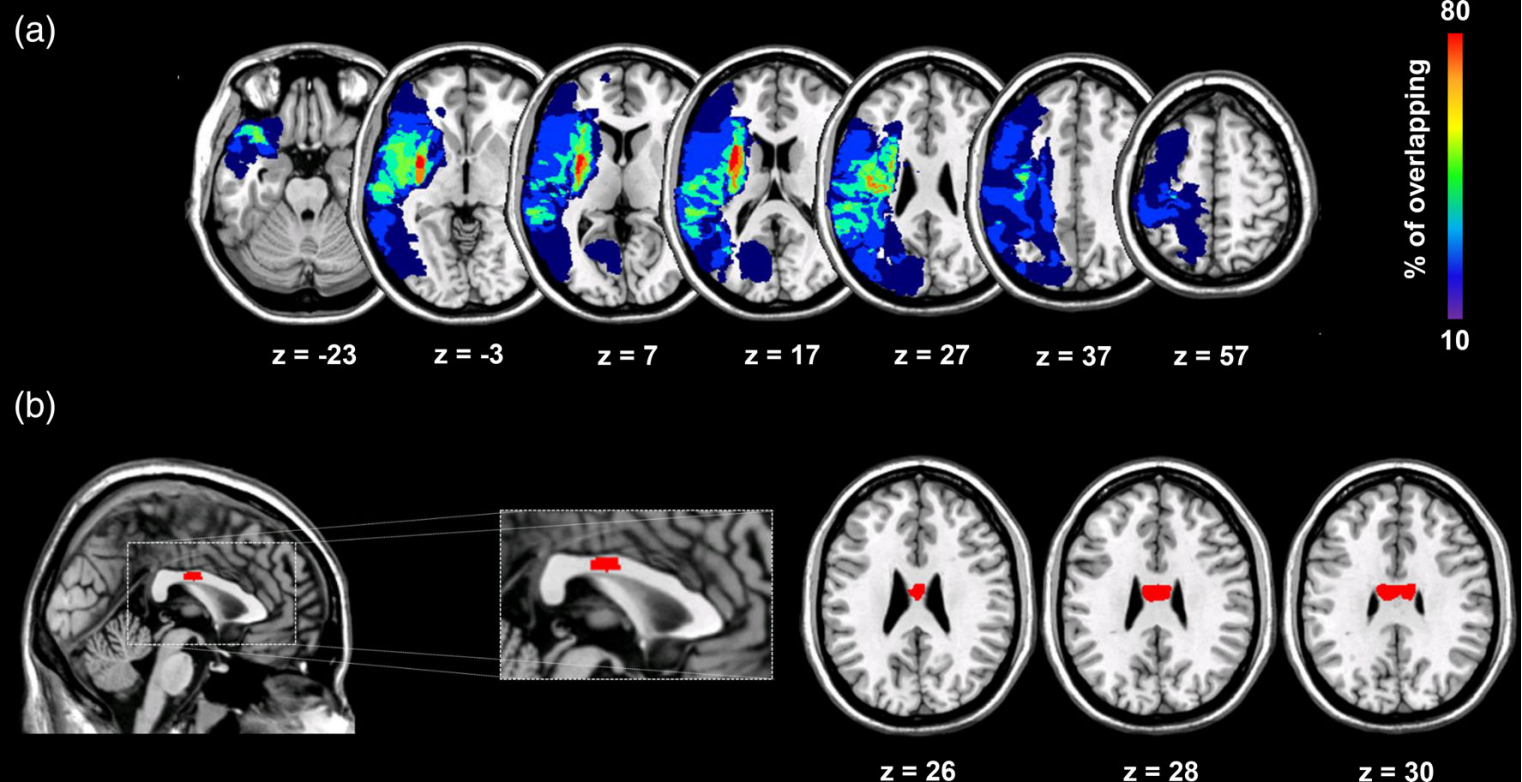
Structural evaluation of stroke patients. Panel (a): Probabilistic lesion maps indicating the percentage of patients with lesion in a given brain area. Panel (b): Tractography of the posterior portion of the midbody section of the corpus callosum overlaid onto a T1‐weighted image in Montreal Neurological Institute space

Nine patients were able to complete the entire structural evaluation with MRI. Seven patients were excluded for the presence of cardiac pacemaker (three patients) or metal implants (four patients) the remaining three patients were not able to complete the entire scanning. Probabilistic lesion maps indicate the percentage of patients with lesion in a given brain area overlaid onto a T1‐weighted image in Montreal Neurological Institute space (Figure 5a). For a descriptive purpose, we swapped left–right orientation of all lesion masks (one patient) located on the LH. Precise lesion location for each patient is reported in Table 1. Figure 5b depicts the tractography of the pCC used for correlation analysis (see below).

### 3.5 Neurophysiological and clinical correlations

**FIGURE 6:**
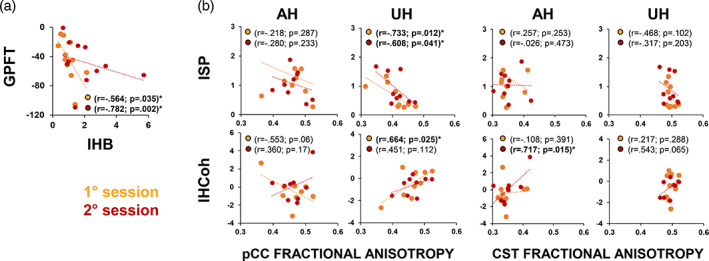
Neurophysiological and clinical correlations. Panel (a): Correlation analysis between grip pinch force test (GPFT) and interhemispheric balance (IHB). Panel (b): Correlation between fractional anisotropy (FA) of the posterior midbody corpus callosum (pCC, Panel (c) left) and of the corticospinal tract (CST, Panel (c) right) with interhemispheric signal propagation (ISP) and interhemispheric coherence (IHCoh)

Correlation conducted on clinical data were significant only for the GPFT scores, which were linearly related to IHB in both the experimental sessions (first: *R* = −.564; *p* = .035; second: *R* = −.782; *p* = .002) (Figure 6a). No significant correlations emerged between corticospinal and cortical measures (all *p*s > .05). Correlations conducted on structural data were significant only for FA of the CC in the posterior midbody section (pCC). Figure 6b depicts the correlations with the pCC (left panel) and with CST (right panel). When analyzing data from the first session, we observed a negative correlation between the FA of the pCC and the ISP after UH stimulation (*r* = −.733; *p* = .012) but not after AH stimulation (*r* = −.218; *p* = .574) (Figure 6b). A significant positive correlation was also observed between the CC volume and the IHCoh in the alpha band after UH stimulation (*r* = .664; *p* = .025) but not after AH stimulation (*r* = −.553; *p* = .122). No significant correlations were observed with FA of the CST (Figure 6b) nor with other sections of the CC. No correlations with MD were significant. After 3 weeks, pCC‐FA still correlated with ISP_UH_ (*r* = −.608; *p* = .041) but not with the M1‐M1 spectral coherence (*r* = .451; *p* = .112).

## 4 DISCUSSION

Here, we provide the first detailed characterization of interhemispheric dynamics in unilateral stroke patients, basing on neurophysiological, structural, and clinical measures. We found that the stimulation of the AH did not result in any interhemispheric suppression of TMS‐evoked cortical activity onto the UH, as normally observable in the healthy brain. Surprisingly, the stimulation of the UH revealed, on the contrary, a preservation of the interhemispheric suppression mechanism, similarly to what we observed in the healthy brain. Then, we computed an index, that is, IHB, reflecting the balance between the two hemispheres, which we recently validated in a large sample of 50 old and young healthy volunteers (Casula et al., 2020). We found an abnormally higher IHB in stroke patients, compared to healthy volunteers, revealing an imbalance between the two hemispheres. Interestingly, we found that IHB was negatively correlated with GPFT, which reflect the difference in strength between the two upper limbs. Thus, our results showed that patients with a more stable IHB, that is, IHB ≈ 1, also showed a smaller difference between the strength of the two upper limbs, that is, GPFT≈0. Finally, we found that patients who had a higher FA of the pCC fibers were the ones who showed (a) a lower ISP from the UH, that is, a higher IHI, and (2) a higher M1‐M1 IHCoh, that is, a stronger interhemispheric connectivity.

So far, TMS studies investigating interhemispheric dynamics after a stroke event, succeeded to test only a minority of patients with recordable MEPs, leading to variable results (Borich et al., 2015; Bütefisch et al., 2008; Cassidy et al., 2015; Dimyan et al., 2014; Stinear et al., 2015). In our corticospinal evaluation, more than 70% of our sample of patients did not show a stable and sufficiently large MEP for the evaluation of IHI nor for the intracortical circuits from the AH. Although this might seem surprising, such high percentage of cases with no recordable MEPs is because we recruited patients with hemiparesis due to subcortical or cortical lesion in the territory of the middle cerebral artery. Previously, TMS studies had to focus mostly on milder subcortical stroke patients with a recordable MEP, to be performed (Borich et al., 2015; Bütefisch et al., 2008; Cassidy et al., 2015; Dimyan et al., 2014; Stinear et al., 2015). On the other hand, by using TMS–EEG, we were able to collect stable measures from both the hemispheres of all patients. Importantly, although a statistical trend can be observable (0.07), the cortical activation of the AH and UH, when directly stimulated, did not differ, in agreement with previous studies (Koch et al., 2019; Pellicciari et al., 2018). On the other hand, as previously observed, cortical activity of both the hemispheres in stroke patients was generally lower compared to HC, likely due to altered excitability in the stimulated neuronal populations and in their cortico‐cortical connections (Pellicciari et al., 2018). So far, only a few studies applied TMS–EEG to evaluate the cortical state following a stroke event (Borich, Wheaton, Brodie, Lakhani, & Boyd, 2016; Gray, Wolf, & Borich, 2017; Manganotti, Acler, Masiero, & del Felice, 2015; Pellicciari et al., 2018). However, these studies were mainly focused on the analysis of cortical activity in the AH without an in‐depth characterization of the interhemispheric interactions. Here, for the first time, we characterized interhemispheric dynamics at different levels, that is, transmission, balance, and connectivity, showing a high specificity and reliability of our measures.

To assess the interhemispheric transmission, we computed the ISP, which represents the propagation of TMS‐evoked activity from the stimulated hemisphere to the contralateral one. ISP has already been used by previous works (Jarczok et al., 2016; Määttä et al., 2017; Voineskos et al., 2010) even if these studies did not verify the sensitivity nor the reliability of this measure. We recently tested ISP in a large sample of healthy volunteers, founding an extremely low intersubject variability among the 50 participants tested (Casula et al., 2020). Although the physiological mechanism underlying ISP has not been fully elucidated, it is likely mediated by transcallosal inhibitory fibers, given its strict correlation with microstructural integrity of callosal fibers (Voineskos et al., 2010), a result that we confirmed in the present study. Along the same lines, we recently observed a linear correlation between ISP and IHI, as measured with MEPs (Casula et al., 2020). This result confirmed the transcallosal origin of the ISP and suggests that it has an inhibitory origin, at least to some extent. To test the ISP sensitivity, we compared stroke patients with a group of HC and tested ICC. HC showed a highly consistent ISP pattern, with a significant suppression of TMS‐evoked activity onto the hemisphere contralateral to TMS, as previously observed in healthy adults (Casula et al., 2020; Määttä et al., 2017) and children (Määttä et al., 2017). In stroke patients, the stimulation of the AH resulted in an inconsistent pattern of interhemispheric transmission with eight patients showing ISP suppression, eight patients showing ISP facilitation and three with no change, that is, ISP between 0.97 and 1.05. Differently, when stimulating the UH, we observed a significant reduction of ISP onto the AH. This trend was highly consistent, being observable in 17 patients out of 19, and followed the same pattern observed in the HC group, being statistically not different. In addition, it is important to note that stimulation of AH in stroke patients produced the largest variability in ISP (*SD* = 0.96) compared to stimulation of UH in patients (*SD* = 0.22) and in HC (left UH: *SD* = 0.26; right UH: *SD* = 0.38). We also observed a larger distribution of the ISP_AH_ values ranging from 0.27 to 3.71, compared to ISP_UH_ in patients (0.27–1.35) and ISP in HC (left UH: 0.28–1.08; right UH: 0.14–1.32). These results demonstrate that ISP is able to discriminate the different pattern of interhemispheric transmission from the AH and UH, regardless the presence of MEP. From a physiological point of view, the inconsistent ISP pattern when tested from the AH, is likely due to an abnormal inhibitory/excitatory activity originating from the lesioned hemisphere (Bütefisch, Netz, Wessling, Seitz, & Hömberg, 2003).

To assess IHB, we computed a ratio between the ISP of the two hemispheres, a measure that we termed IHB. This measure offers a direct measure of the IHB and has been used for the first time by a recent study of our group, which found a high consistency of this measure both within‐ and between‐subjects (Casula et al., 2020). Here, we observed that HC showed a similar interhemispheric dynamic from both the hemispheres, that is, similar ISP, resulting in an IHB ≈ 1. In stroke patients, the different ISP pattern of the two hemispheres resulted in an extremely high IHB with a large intersubject variability. Indeed, in patients, IHB values were more variable (2.45) than in HC (0.87) with a larger distribution ranging from 0.64 to 9.15, compared to HC (0.26–3.36). Importantly, we also observed a negative correlation between GPFT and IHB, meaning that patients who had a minor difference between the strength of the two upper limbs also showed a more stable balance between the two hemispheres. When retested after 3 weeks, the correlation was confirmed with, as expected, no significant changes in the GPFT score, since our stroke patients were in a chronic stage. From a functional point of view, a higher score in the GPFT is indicative of a strength recovery of the affected hand and when its cortical representation is stimulated with TMS of the AH, the evoked activity follows a dynamic similar to the one observed after stimulation of the UH. These results provide a direct support to the so‐called interhemispheric imbalance model (Boddington & Reynolds, 2017) according to which, in healthy condition, each hemisphere inhibits the other equally, whereas in stroke patients IHI from the AH is decreased, with a lower excitability in the perilesional tissue. Other models, such as the bimodal balance–recovery model, postulate that the contribution of interhemispheric imbalance in stroke recovery varies according to the so‐called structural reserve (Boddington & Reynolds, 2017; di Pino et al., 2014). Structural reserve is defined as the quantity of strategic neural pathways and relays that are spared by the lesion. Patients with high structural reserve often achieve better functional recovery. In such cases, the balance of activity between the two hemispheres tends toward the previous equilibrium, whereas persistence of interhemispheric imbalance is a predictor of worse outcome. On the other hand, in cases in which structural reserve is smaller (i.e., patients with more‐severe impairment) persistence of interhemispheric imbalance could be important to promote vicarious activity of the UH, allowing some compensatory plasticity. Our data were collected in a limited sample of patients and thus does not allow testing the different conditions according to the level of structural reserve. Further studies are needed to elucidate the potential interactions between interhemispheric imbalance and structural reserve in stroke patients using TMS–EEG.

To assess interhemispheric connectivity, we computed spectral coherence between the two stimulated M1s, that is, IHCoh. When tested with TMS, spectral coherence represents a direct index of functional connectivity given that the stimulation is able to reset the ongoing rhythmic EEG activity. Previous studies testing spectral coherence in a group of chronic stroke patients, found that TMS‐evoked beta activity was associated with transcallosal inhibition (Borich et al., 2016) and motor contraction (Palmer et al., 2019). The ad hoc investigation of beta activity was justified by the authors with different lines of evidence showing that this frequency is observable over M1 at rest and during sustained isometric contractions (Baker, Olivier, & Lemon, 1997; Irlbacher, Brocke, Mechow, & Brandt, 2007). In the present study, to select our frequency of interest we exploited the capacity of TMS to evoke the so‐called natural frequency that is the predominant frequency at which the activity of a specific area oscillates (Rosanova et al., 2009). We found that the stimulation over M1 induced a sustained activity lasting ≈200 ms mainly in the alpha range, with a maximum power centered between 10 and 11 Hz, as we previously observed both in stroke patients (Koch et al., 2019; Pellicciari et al., 2018) and in healthy volunteers (Casula et al., 2016). Notably, the same natural frequency was observable both in HC and in stroke patients and, more importantly, in both the hemispheres. In addition, although some differences were appreciable, the power in the natural frequency of the two hemispheres was not different. Along the same line, we did not observe any difference between the two groups in M1‐M1 IHCoh. Several reasons could explain this lack of difference: first, it could be conceivable that these indexes are not sensitive to interhemispheric dynamics, as also revealed by the low reliability of this measure; second, our sample size was too small; third, interhemispheric oscillatory activity of stroke patients and HC does not differ at rest, as previously suggested (Borich et al., 2016).

To further verify the specificity of our measures, we tested whether TMS–EEG interhemispheric measures were correlated with corticospinal and structural data. As previously observed (Casula et al., 2014), we did not observe any significant correlation between TMS–EMG and TMS–EEG measures presumably because of their different physiological origin. Indeed, while MEPs reflect the excitability of the whole CST, TMS‐evoked EEG activity results from post‐synaptic potentials following the neuronal depolarization caused by TMS (Ilmoniemi et al., 1997). Analysis of interhemispheric and structural data showed that the FA of the pCC was directly correlated with M1‐M1 IHCoh and inversely correlated with ISP when these measures were evoked from the UH, but not when evoked from the AH. These two results support the validity of M1‐M1 IHCoh as a marker of interhemispheric connectivity. Indeed, higher FA of callosal fibers, through which the communication between the two M1s occurs, was associated with a more efficient interhemispheric connectivity (i.e., higher IHCoh) and transmission (i.e., lower ISP). It is important to note that our correlations were found as significant only with the FA of the pCC, in agreement with previous studies showing that this specific region is responsible for the interhemispheric connection of motor (Wahl et al., 2007) and nonmotor areas (Koch et al., 2011). Notably, no significant correlations were observed when we tested the correlations with FA of the CST of both sides, excluding an aspecific role of the corticospinal fibers in interhemispheric dynamics.

Finally, to ensure the repeatability of our measures we retest our patients sample with TMS–EEG after 3 weeks. Analysis of test–retest reliability showed that ISP and IHB were highly reliable in healthy volunteers and that with a sample size of 15 we will observe an SDC of 0.1. When tested in stroke patients, the reliability was significantly lower and higher sample size (50 for ISP and more than 60 for IHB) are needed to observe an SDC of 0.1. This might be due to the higher variability observed in the cortical reactivity to TMS, especially when testing the lesioned hemisphere. Importantly, correlation analyses between cortical, and clinical and structural data were highly reproducible, a result that supports their use in clinical evaluation. Finally, analysis of ICC showed that subjects in the same group resemble each other in terms of ISP and IHB. This result is important because it shows that ISP and IHB are reliable measures in distinguish healthy and pathological interhemispheric dynamics. Differently, our results showed a low ICC reliability of IHCoh, a result that can explain the absence of correlation with structural data after 3 weeks.

The physiological nature of the novel interhemispheric TMS–EEG indexes presented here is not completely understood. However, we recently found a strict correlation between our TMS–EEG indexes and the traditional TMS–EMG of IHI, that is, IHI, tested with two coils over the M1 of the two hemispheres (Casula et al., 2020). From a physiological point of view, it is known that the mechanism of IHI is mediated the fibers connecting the two hemispheres, namely the callosal fibers. Previous studies conducted in animals, demonstrated the GABAergic inhibitory nature of most of the callosal projections, which are responsible for the inhibition of the contralateral hemisphere (Chen, 2004; Irlbacher et al., 2007). Specifically, early and later IHI, are responsible for the early and later IHI, respectively (Irlbacher et al., 2007; Müller‐Dahlhaus, Liu, & Ziemann, 2008). Starting from these considerations, we speculate that ISP reflects, at least to some extent, the IHI mechanism mediated by the callosal fibers. In the present study we could not confirm the correlation between the well‐established TMS–EMG‐based measure of IHI and the novel TMS–EEG measures in stroke patients, given the impossibility to evoke MEP from the AH; however, our results are in line with previous finding of a disrupted mechanism of IHB in these patients.

A main limitation of the present study is the presence of both left‐sided (6 patients) and right‐sided (13 patients) lesions that lead us to collapse all the ipsilesional the AHs in one side, with a reduction of the spatial accuracy. Another limitation of the study lies in the impossibility to accurately estimate the intensity of stimulation for the AH in some patients. However, we demonstrated that this factor did not affect our results. Indeed, our results were also confirmed in a subgroup of stroke patients with an evocable MEP from the AH. Moreover, only a low number of patients completed the clinical and the structural evaluation, thus our conclusions regarding the correlation analysis should be considered as exploratory. Finally, it is conceivable that the suppression of TMS‐evoked activity is produced, at least to some extent, by a degradation of the activation spreading through biological tissue (Määttä et al., 2017). However, we tend to exclude this interpretation for a number of reasons: (a) when ISP is tested in the same hemisphere, it is greater than when tested between the two hemispheres; (b) adults showed a larger ISP compared to children, who have smaller heads; and (c) intensity of stimulation does not affect ISP. Another alternative hypothesis could be that suppression of TMS‐evoked activity depended on the different cortical activation per se, and not on an interhemispheric mechanism. However, also this hypothesis is excludable for two reasons: (a) when directly stimulated the cortical activation of AH and UH did not differed and (b) cortical activation of the UH in stroke patients was lower compared to the activation in HC, despite its mechanisms of interhemispheric suppression of TMS‐evoked activity was stable and reproducible.

In conclusion, the main contribution of this study lies in the proposal of new TMS–EEG indexes of interhemispheric dynamics in stroke patients. These measures show high reliability in distinguishing healthy and pathological interhemispheric dynamics and, more importantly, can be evoked even in absence of a stable MEP, which is often unreliable in stroke patients. These findings could be of high relevance from a clinical point of view. Indeed, a part from the intrinsic limitations of the technique, for example, the time of acquisition and analysis of the signal, TMS–EEG provide noninvasive and in vivo measures of the cortical state during the evolution of the cerebral reorganization right after a stroke event, as we recently demonstrated in acute stroke patients (Pellicciari et al., 2018).

## CONFLICT OF INTEREST

The authors declare no conflicts of interest.

## AUTHOR CONTRIBUTIONS

**Elias Paolo Casula**: Conception and design of the work, data analysis, manuscript writing, revision of the work. **Maria Concetta Pellicciari**: TMS–EEG data analysis. **Sonia Bonnì**: Data collection, patient's recruitment. **Barbara Spanò**: MRI data analysis. **Viviana Ponzo**: TMS–EEG and TMS–EMG data collection. **Ilenia Salsano**: MRI data collection and analysis. **Giovanni Giulietti**: MRI data analysis. **Alex Martino Cinnera**: Functional and behavioral data collection. **Michele Maiella**: TMS–EEG data collection and analysis. **Ilaria Borghi**: TMS–EEG data collection and analysis. **Lorenzo Rocchi**: Manuscript writing, revision of the work. **Marco Bozzali**: MRI data analysis, revision of the work. **Fabrizio Sallustio**: Patient's recruitment, revision of the work. **Carlo Caltagirone**: Revision of the work. **Giacomo Koch**: Conception and design of the work, revision of the work.

## Data Availability

The data that support the findings of this study are openly available in Google drive (https://drive.google.com/open?id=1te3HavEq1q7aGFM1wCyVRkVQzXDYbEM).
